# Quality of life of colorectal cancer survivors in a Ghanaian population

**DOI:** 10.1186/s13104-019-4817-8

**Published:** 2019-11-29

**Authors:** Joseph Yorke, Emmanuel Acheampong, Emmanuella Nsenbah Batu, Christian Obirikorang, Francis Agyemang Yeboah, Evans Adu Asamoah

**Affiliations:** 1 Department of Molecular Medicine, School of Medical Science, Kwame Nkrumah University of Science and Technology (KNUST), Kumasi, Ghana; 2 Department of Surgery, Komfo Anokye Teaching Hospital, Kumasi, Ghana; 3 School of Medical and Health Sciences, Edith Cowan University, Joondalup, WA Australia

**Keywords:** QoL, Colorectal cancer, EORTC QLQ-C30, EORTC QLQ-CR29

## Abstract

**Objective:**

We collected data to evaluate the quality of life of patients who have survived between one and 8 years from the diagnosis of colorectal cancer.

**Data description:**

We collected quality of life (QoL) data from colorectal patients who were diagnosed between 2009 and 2015 at the Komfo Anokye Teaching Hospital (KATH) and have survived until January 2017. The dataset consists of patients’ demographic data, clinicopathological characteristics, and QoL data. The validated QoL instruments for data curation was an adopted version of the European Organization for Research and Treatment of Cancer (EORTC) QLQ-C30 and the EORTC QLQ-CR29. The QLQ-C30 was a 30-item general cancer instrument with 5 functional subscales, and 9 symptom subscales, whereas the QLQ-CR29 was a 29-item scale that consisted of 3 functional QOL subscales and 14 symptom subscales, that are associated with colorectal cancer and its treatment. The QoL instrument was coded such that higher scores indicated increased function and better QoL, and higher symptom scores represent worse symptoms.

## Objective

Colorectal cancer (CRC) from the global perspective is the third most common malignant neoplasm but was considered to be rare within the African context [1, 2]. However, recent accumulating evidence has shown that numerous African countries which were traditionally recognized as low-risk countries [3, 4] including Ghana [5], has reportedly increased the rate of CRC. These trend has previously been confirmed in our previous work [6] at a major teaching hospital in Ghana. CRC in these countries represents about 10–50% of all malignant tumours and has a characteristic unique pattern with an early age of onset and mostly left-sided tumours [6, 7].

Whereas an increase in survival rates is clearly a great accomplishment, there are unintended negative consequences with treatments that can potentially reduce the QoL [8, 9]. Colorectal cancer patients may suffer long-lasting pain and reductions in functional and social well-being irrespectively of the type of treatment including surgery, radiation therapy, and systemic chemo-and targeted therapy [10]. Therefore, we explored the extent to which health-related quality of life is affected by CRC and identified key areas that merit further attention to improve the quality of survival after CRC is being diagnosed and treated. These effects are explored through the analysis of survey questions answered by survivors of colorectal cancer relating to their QoL. The current data on QoL in CRC patients may improve our understanding of how cancer and its therapy influence the patients’ lives, and how to adapt appropriate treatment strategies. Part of the results based on this data has been published in PLOS ONE [11].

## Data description

We did our data collection in two parts. First, we did a retrospective review of the case files of all CRC patients diagnosed and managed at KATH from 2009 to 2015 and have survived till January 2017, from the Medical records unit of the surgery department and the Oncology Department. We analyzed for information on demographics, clinical and pathological variables including histological type, grade of tumour and staging based on the TNM. The type of treatment and follow-up were also analyzed. Information on age at diagnosis, gender, tumour location, pathological type of tumour, treatment modality, family history of CRC, and metastasis were also reviewed, and the dataset is shown in Table 1. Secondary, all patients whose information was reviewed were contacted through phone calls, their identity confirmed with age, name, and hospital identification number and visited by the research team for interview. In total, 220 cases were confirmed and included after obtaining their verbal and written informed consent to partake in the study.

**Table 1:** Overview of data files/data sets

Label	Name of data file/data set	File types (file extension)	Data repository and identifier (DOI or accession number)
Data file 1	Table for comparison of QOL based on primary cancer site	MS Excel file (.xlsx)	10.6084/m9.figshare.9958673.v1
Data set 1	Colorectal cancer data set (Quality of life)	MS Excel file (.xlsx)	10.6084/m9.figshare.9958670.v4

The instrument used to assess QoL in this study were the European Organization for Research and Treatment of Cancer (EORTC) QLQ-C30 and the EORTC QLQ-CR29. The QLQ-C30 was an overall cancer instrument which contains a 30-items that assess global QOL; 5 functional subscales (emotional, cognitive, social, and physical role), and 9 symptom subscales (nausea/vomiting, fatigue, appetite loss, diarrhoea, sleep disturbance, pain and financial influence) [12]. The EORTC QLQ-CR29 consist 29-items that assess three functional QOL items (weight anxiety and body image) and (14) symptom items (mucus and blood in stool, frequency of urination and stool, dysuria, pain in the abdomen and buttock, feeling bloated, urinary and faecal incontinence, bloated feeling, dry mouth, loss of hair, trouble with taste, sore skin, and flatulence) that are related to CRC and its treatment [13]. For both QLQ-C30 and QLQ-CR29, the responses were scored on a Likert scale of 4 response categories. Higher functional and global QoL domain scores indicated increased function and better QoL, and a higher symptom score represents worse symptoms. The dataset for responses to QLQ-C30 and QLQ-CR29 has been shown in Table 1.

## Limitations

The data reflect specific patient population reporting to KATH. Thus, making it an institutional-based study but not a population-based study. Also, we assumed, those who die before a year are likely to have more advanced disease and more co-morbidities than those who survive and are likely to report low QoL, however, our data did not capture such patients. No information was available from respondents on their QoL prior to being diagnosed with CRC. In the same way, there was no information available for non-cancer controls of age, sex and socio-economic status matched population, which is considered a major limitation of the study.

## Data Availability

The data described in this data note is available and can be freely and openly accessed [14, 15]. Please see Table 1 and reference list for details and links to the data.

## Abbreviations

CRC: colorectal cancer
EORTC: European Organization for Research and Treatment of Cancer
QoL: quality of Life
